# Is the Retinal Vasculature Related to *β*-Peripapillary Atrophy in Nonpathological High Myopia? An Optical Coherence Tomography Angiography Study in Chinese Adults

**DOI:** 10.1155/2018/7895238

**Published:** 2018-09-30

**Authors:** Jiao Sun, Jialin Wang, Ran You, Yanling Wang

**Affiliations:** Department of Ophthalmology, Beijing Friendship Hospital Affiliated to Capital Medical University, Beijing, China

## Abstract

**Purpose:**

The association between *β*-peripapillary atrophy and the retinal vasculature in nonpathological high myopia is unclear. The aim of this study is to investigate whether *β*-peripapillary atrophy contribute to the changes of the retinal vasculature using optical coherence tomography angiography.

**Methods:**

In a cross-sectional study, one hundred and thirty eyes with nonpathological high myopia were included. *β*-peripapillary atrophy was analysed using Image J software based on fundus photographs. A 3.0 × 3.0 mm^2^ grid and a 4.5 × 4.5 mm^2^ grid were used to scan parafoveal and peripapillary regions using optical coherence tomography angiography, respectively. Vessel density and fractal dimensions of the retina and foveal avascular zone were analysed and quantified using en face projection images. Correlations between the vascular density, foveal avascular zone, and *β*-peripapillary atrophy were determined.

**Results:**

Using multivariate analysis, *β*-peripapillary atrophy was negatively correlated with the vessel density in radial peripapillary capillaries (*p*=0.002) even after adjusting for other variables. This relationship was also confirmed in the macula (superficial retinal plexus: *p* < 0.05; deep retinal plexus: *p* < 0.05). The vessel densities in the nasal and inferior sectors were more strongly correlated with *β*-peripapillary atrophy.

**Conclusions:**

There was a negative correlation between *β*-peripapillary atrophy and the retinal vasculature in highly myopic eyes, especially in radial peripapillary capillaries and deep retinal plexus. *β*-peripapillary atrophy can be visualized and is a convenient structural feature that can benefit the early diagnosis and detection of chorioretinal atrophy in high myopia.

## 1. Introduction

The prevalence of myopia has markedly increased over the past three decades, especially in China [1]. In myopic eyes, there are morphological changes in the optic disc, such as *β*-peripapillary atrophy (*β*-PPA), tilt, and rotation [2]. The myopic *β*-PPA, also known as the optic disc crescent, is a white area with a well-defined boundary and visible sclera due to uncovering of the retinal pigment epithelium (RPE), a common structure in myopic eyes [3]. One study demonstrated that myopic *β*-PPA eyes showed a thinner ganglion cell-inner plexiform layer and macular thickness than myopic eyes without *β*-PPA [4]. Another study found that the quadrantal alterations in myopic eyes were uneven, with the greatest changes noted in the inferior nasal sector [5]. Moreover, Wang and associates [6] found a decrease in peripapillary perfusion in highly myopic eyes and inferred that *β*-PPA may play a role in the decreased peripapillary blood flow in myopic eyes. Therefore, we hypothesized that *β*-PPA might be associated with macular and peripapillary perfusion. Additionally, as *β*-PPA is a common finding in normal-tension glaucoma (NTG) and the pathogenesis between myopia and NTG remains unclear, focusing on a potential shared pathway between the retinal vasculature and NTG could improve our understanding of the pathophysiology and expand therapies for each condition.

Optical coherence tomography angiography (OCTA) is a novel quantitative method used to analyse the retinal microvasculature. Previous studies using different imaging modalities have demonstrated a reduction in retinal and choroidal perfusion in high myopia. In addition, a growing body of evidence suggests that vascular dysfunction is a complication of myopia [3, 7, 8]. Thus, it is important to study retinal perfusion in myopic eyes because this information is helpful for the early diagnosis and monitoring of chorioretinal atrophy in eyes with high myopia [9].

The aims of the present study were to quantify vasculature parameters using OCTA to determine whether retinal vasculature is associated with *β*-PPA and to characterize differences in *β*-PPA with macular perfusion in each sector in highly myopic eyes.

## 2. Methods and Materials

### 2.1. Participants

**Figure 1:**
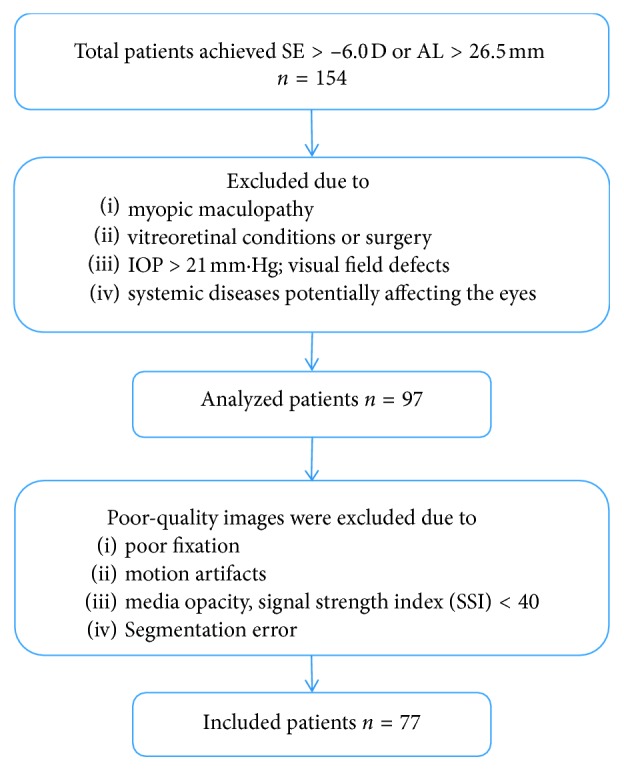
Study population. SE = spherical equivalent; AL = axial length.

Patients from the Beijing Friendship Hospital (Beijing, China) were recruited. This cross-sectional study was approved by the Ethics Committee of the Beijing Friendship Hospital (Beijing, China) and was conducted in accordance with the ethical standards stated in the Declaration of Helsinki. Written informed consent was obtained from all examined patients and volunteer participants prior to OCTA imaging. Each subject underwent a complete ocular examination, including best-corrected visual acuity (BCVA), intraocular pressure (IOP) by automatic tonometer, slit-lamp examination, fundus colour photography, and axial length (AL) measurement by optical biometry (IOL Master; Carl Zeiss Meditec, Jena, Germany). Subjects with a refraction of greater than 6 diopters (D) or ALs longer than 26.5 mm were included in this study. The exclusion criteria were myopic maculopathy, any prior history or clinical evidence of vitreoretinal conditions or surgery, IOP > 21 mmHg, visual field defects, and systemic diseases potentially affecting the eyes (Figure 1).

### 2.2. Image Acquisition and Analysis

**Figure 2:**
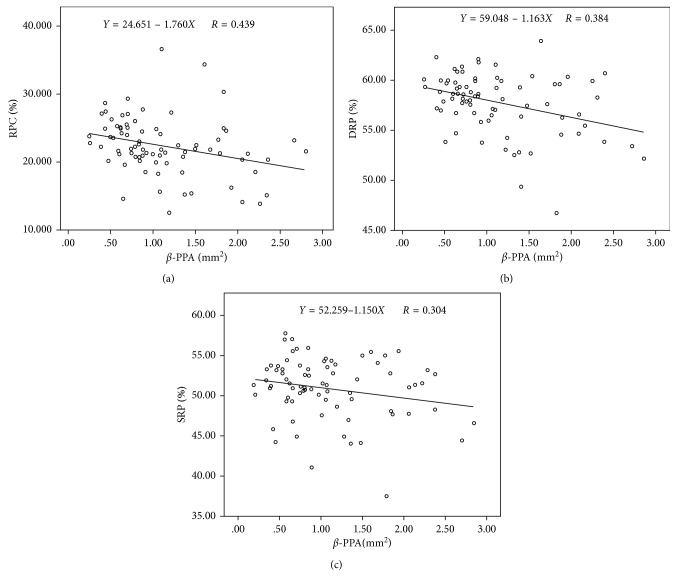
Correlation analyses between vessel density and *β*-peripapillary atrophy in the retina. (a) Radial peripapillary capillaries. (b) Deep retinal plexus. (c) Superficial retinal plexus.

The OCTA instrument, RTVue XR Avanti with AngioVue (Optovue Inc., Fremont, California, USA), was used at a scanning speed of 70,000 A-scans per second by a single operator (JS). The scan protocol examined a 3.0 × 3.0 mm^2^ area focused on the macula and a 4.5 × 4.5 mm^2^ area focused on the optic disc and obtained a horizontal priority (*X*-scan) and a vertical priority (*Y*-scan) in approximately 2.9 s for each of the two raster scans. The RTVue instrument was used to segment superficial and deep inner retinal vascular plexuses (Figure 2). The superficial retinal plexus (SRP) was segmented from the outer boundary of the inner limiting membrane (ILM) to the outer boundary of the inner plexiform layer (IPL), which extends from 3 *μ*m below the ILM to 15 *μ*m below the IPL. The deep retinal plexus (DRP) was segmented from the outer boundary of the IPL to the outer boundary of the outer plexiform layer (OPL), which extends from 15 to 70 *μ*m below the IPL. The split-spectrum amplitude decorrelation angiography (SSADA) algorithm was used to segment the vessels. The superficial retinal layer comprises ganglion cell and inner plexiform layers, whereas the deep retinal layer comprises inner nuclear and outer plexiform layers. These layers encompass the entire retinal vascular network. Furthermore, OCTA images of the superficial and deep retinal layers were divided into 5 parts: fovea, nasal, temporal, inferior, and superior sectors. The resolution of the exported OCTA images was 304 × 304 pixels. The motion correction technology (MCT) function was used to correct the horizontal and vertical scans. Because the magnification is different in myopic eyes, the imaging sampling density used in myopic eyes must be lower than that used in normal eyes [10]. Therefore, images obtained from highly myopic eyes were corrected for magnification using Bennett's formula. The RTVue instrument was also used to measure the retinal nerve fiber layer (RNFL) thickness and cup-to-disc ratio (CDR) from the OCT B-scans. Two independent examiners (XMM, FZ) reviewed the images. Poor-quality images were excluded based on the following criteria: (1) evidence of poor fixation, including a double vessel pattern and motion artefacts; (2) the presence of motion artefacts that could not be corrected by MCT; (3) media opacity, as marked by shadowing or obscuration of the vessel signal in the field of view or a signal strength index (SSI) of less than 40; and (4) a segmentation error in defining vascular layers (Figure 1).

**Figure 3:**
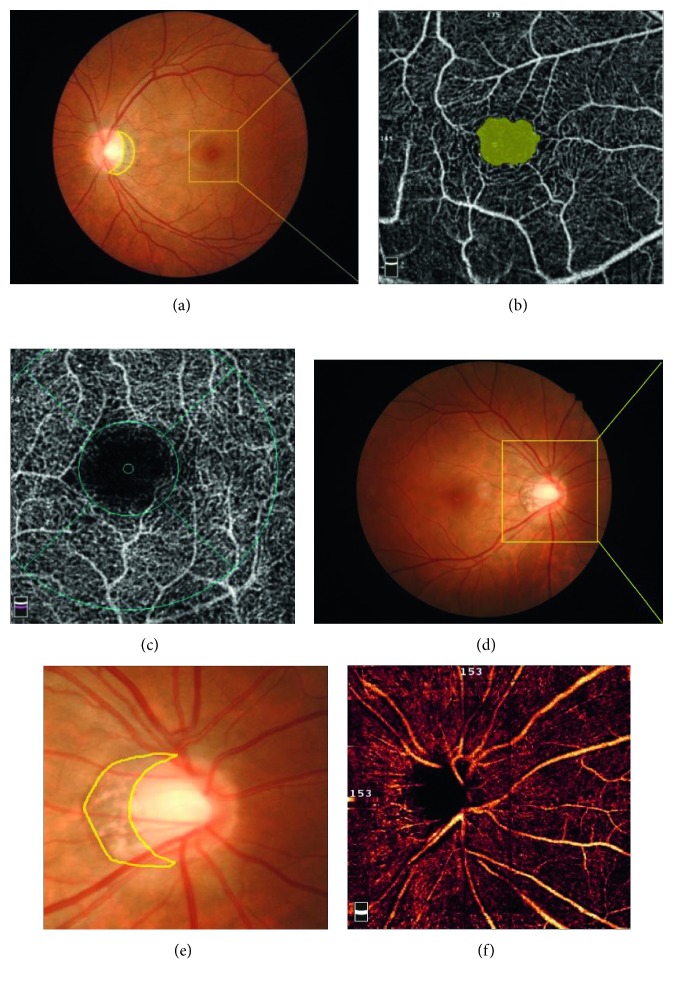
Images of *β*-peripapillary atrophy in a fundus colour paragraph (a, d, e), foveal avascular zone and vessel images of the superficial retinal plexus (b), deep retinal plexus (c), and radial peripapillary capillaries (f) using optical coherence tomography angiography in areas of 3.0 × 3.0 mm^2^ and 4.5 × 4.5 mm^2^.

High-resolution digital colour fundus photographs were taken using a digital retina camera (Kowa Nonmyd WX; Kowa Company Ltd., Japan). *β*-PPA is defined as the area in a fundus colour photograph characterized by a marked atrophy of retinal photoreceptors, RPE, and the choriocapillaris, along with the distinct visibility of large choroidal vessels and the sclera (Figure 3). When there was disagreement between the graders, we outlined the area with the help of OCT.

OCTA data were analysed using Optovue software (RTVue XR version 2016.2.0.35). The foveal avascular zone (FAZ) area for each superficial plexus image was found and measured using the built-in nonflow automatic measurement tool of the AngioVue review software. Image processing of the radial peripapillary capillary (RPC) vascular density and *β*-PPA area measurements were performed using the public domain software Image J, version 1.50i (National Institutes of Health, Bethesda, Maryland, USA) (Figure 3). Binary images of the vascular networks were created using an automated thresholding algorithm. Vessel density was defined as the percentage area occupied by blood vessels, with the blood vessels being defined as pixels having values above the threshold level [11]. To assess the reproducibility of the technique, two examiners (XMM, FZ) measured each image 3 times.

### 2.3. Statistical Analysis

Statistical analysis was performed using a commercially available statistics software programme (SPSS for Microsoft, version 24.0; IBM/SPSS, Chicago, Illinois, USA). First, we calculated the means and standard deviations of the main outcome parameters. Second, we carried out multivariate regression models, modelling the vasculature parameters as the dependent variable, and all parameters that were associated with the vasculature parameters as independent variables, taking a *p* value < 0.05 as the cutoff value. Third, we performed a linear regression of potential associations between *β*-PPA and macular vessel density in each sector. Finally, we calculated the standardized regression coefficient beta and the nonstandardized regression coefficient *B* together with its 95% confidence interval (CI).

## 3. Results

### 3.1. Demographics

Among the 97 participants recruited, 20 were excluded from the analysis because of poor signal quality (SSI < 45) or blink artefacts. A total of 130 eyes from 77 participants with nonpathological high myopia were analysed in this study. The mean age at the initial visit was 35.24 ± 8.45 years, and the mean spherical equivalent (SE) refractive error was −10.03 ± 3.57 D. The demographics of these participants are shown in Table 1.

### 3.2. Association between *β*-PPA and RPC Parameters

Using linear regression analysis, *β*-PPA was shown to be negatively associated with the RPC (Figure 2(a), Table 2, *p* < 0.001). After adjusting for the compounding factors of age, IOP, CDR, and RNFL thickness, the parameter AL was still significantly negatively correlated with RPC (*β* = −1.472, *p*=0.002) (Table 2). Specifically, a 1.0 mm^2^ increase in *β*-PPA was associated with 1.472% lower in RPC density.

### 3.3. Association between *β*-PPA and Macular Perfusion Parameters

Using linear regression analysis, *β*-PPA was shown to be negatively associated with SRP and DRP (Figures 2(b) and 2(c), Table 3, *p* < 0.001 and *p*=0.005, respectively, for DRP and SRP). An increase in *β*-PPA by 1 mm^2^ was associated with a decrease in the vessel density of DRP by 1.163%, whereas the SRP lessened by 1.150%. Using multiple linear regression analysis, *β*-PPA was shown to be negatively associated with DRP in model 2 when we adjusted age and gender (Table 3, *β* = −0.874, *p* < 0.05); when we added the factors of IOP, CDR, and RNFL thickness, *β*-PPA is still significantly correlated with SRP (Table 3, model 3: *β* = −0.836, *p* < 0.05). After adjusting AL in model 4, we found that the *p* value was very closely approaching the conventional significance level (*p*=0.059, when we take a *p* value < 0.05 as the cutoff value).

### 3.4. Association between *β*-PPA and Macular Perfusion Parameters in Each Sector

Furthermore, we concluded the results of the regression analysis associating *β*-PPA with macular perfusion parameters in different sectors. As shown in Table 4, after we had adjusted the factors of age, gender, IOP, CDR, RNFL thickness, and AL, *β*-PPA is still significant negatively correlated with DRP in the nasal sector (*β* = −1.029, *p* < 0.05). Moreover, the *p* value of the DRP inferior sector is approaching the conventional significance level, although not reaching significance (*p*=0.083). In SRP, the *p* value in the nasal sector nearly reached a significance level (*p*=0.090). The nonsignificant results (with *p* values larger than 0.05) in Table 4 may be explained by a single research center in this study. Further studies in multiple clinical research centers are needed to clarify the associations between *β*-PPA and retinal perfusion.

## 4. Discussion

The present study revealed a negative correlation between *β*-PPA and retinal vascular densities in the macula and RPC (Figure 2), although there was no correlation between *β*-PPA and foveal vascular density and FAZ. In the fractal analysis, this correlation was more significant in the nasal and inferior regions. Earlier work demonstrated that vessel density was reduced in high myopia compared with that in healthy subjects [12]. However, these studies did not consider *β*-PPA. To our knowledge, our analysis is the first to study the associations of *β*-PPA with the retinal vasculature and to characterize differences in *β*-PPA with macular fractal perfusion in highly myopic eyes.

The retina has three levels of blood supply (plexuses): the RPC plexus, superficial plexuses, and deep plexuses [13]. Half of the inner retina receives blood supply from three of these vascular plexuses, whereas choriocapillaries provide blood supply to the outer half of the retina and macula, and these are derived from the posterior ciliary artery (PCA) [14]. Histological findings have shown that photoreceptors, RPE, and choroid fall short and suffer even partial or complete loss at the temporal atrophic area of the crescent in myopia. Due to stretching forces and retinal degeneration, the vessels in *β*-PPA become straighter and thinner [15]; this may damage the endothelial cells and thus reduce the concentration of vascular endothelial growth factor (VEGF). The reduction in VEGF may also contribute to the possible loss of capillary networks [16, 17]. The chloride channels in RPE can regulate the choroid vasculature [18, 19]. Aakriti Garg and associates [20] showed that choroidal thinning is associated with *β*-PPA. Peripapillary intrachoroidal cavitation (PICC) often appears in myopic eyes with *β*-PPA, and patients with PICC have larger *β*-PPA than those without PICC [21]. Furthermore, *β*-PPA in children is associated with extreme peripapillary thinning [22]. Chui and associates [23] reported that retinal stretching may not mirror scleral growth, and their results suggest that there could be slippage within the retina during eye growth. This may be the reason for retinoschisis and thus for the reduction in macular perfusion. Because of the separation tendency with the choroid, the retina cannot obtain enough nutrition. Moreover, thinning of the parafovea and macular ganglion cell-inner plexiform layer occurs in myopic eyes with *β*-PPA [4]. The thinning of the tissue may cause reduced oxygen demand, and as a result, blood circulation may decrease. Previous studies have demonstrated a reduction in the SCP and DCP of the macula in highly myopic eyes, and our results show that the vessel density parameters in both layers were correlated to *β*-PPA. Thus, it can be assumed that the macula is involved in the pathological process at the onset of disease due to the correlation with *β*-PPA.

AL appears to be significantly associated with both *β*-PPA [23] and decreased macular vasculature [24], and the latter two variables are affected by multiple factors. However, myopic disc changes are not always accompanied by axial elongation, which varies among individuals [25], suggesting that *β*-PPA also plays an important role in vasculature changes. In our study, the retinal vasculature parameters were all associated with AL and *β*-PPA. The AL was a confounder in the relationship with *β*-PPA and macular vasculature, while *β*-PPA was independently correlated with the RPC vessel density. Due to the direction of the traction force as AL is elongated, the macula is more strongly affected by AL than the optic disc.

Additionally, previous studies have reported an association between glaucoma and myopia; the mechanism by which myopia increases the risk of glaucoma remains unclear, and an understanding of myopia's role in glaucoma is important. *β*-PPA is a very common and characteristic structure in high myopia and glaucoma. Macular vessel density is decreased in glaucoma and high myopia and is correlated with the extent of disease. A previous study demonstrated that OCTA has the diagnostic value for determining macular vascular density for the early detection of glaucoma [14]. In patients with NTG, eyes with *β*-PPA demonstrated a significantly greater prevalence of retina pseudodrusen (RPD) than eyes without *β*-PPA [20]. A number of studies have reported an association between ocular circulation disorders and myopic morphological change. In the macular area, the GCC is thinner in high myopia, and this can be attributed to the vascular decline. In the peripapillary area, the reduced peripapillary perfusion can lead to the atrophy or lesion of the optic nerve head, RNFL thinning, and visual field defects. Together, these factors can increase the risk of NTG in high myopia. However, whether the reduced vascular perfusion in high myopia is a cause or result of NTG is not clear. Elucidating the effect of myopia on ocular circulation can lead to improved knowledge of the pathogenesis of myopia. However, these correlations require further study.

We observed that the fractal dimension representing vascular density in the whole retina was slightly higher than that in each of the individual layers. Moreover, the strength of the correlation was different in the fractal dimension. Our study further showed that the nasal and inferior sectors were more strongly related than other sectors in the SCP and DCP. This relationship could contribute to the direction of *β*-PPA and the structures related to it. *β*-PPA normally presents temporally or inferotemporally to the disc and must be differentiated from the congenital tilted disc, which normally occurs inferiorly [26]. The PICC is more easily found in the temporal and inferior sectors [21], and the RNFL is thinner in the temporal sector of the optic disc. Inferior subfoveal scleral thickness was lower than that for other regions around the posterior pole. However, further investigation is needed to reveal the pathogenesis underlying the relationship identified in this study. Furthermore, the vessel density in the nasal and inferior sectors may serve as a sensitive indicator of myopic retinopathy, thus explaining the mechanism of the myopia process.

Our study demonstrated that the FAZ of the fovea was not correlated with *β*-PPA. This might occur because most of the oxygen supplied to the retina from the FAZ is derived from the choroidal vessel rather than the retinal circulation, and changes in the FAZ may not be evident in the early stages of high myopia. Hence, we did not observe a correlation between the FAZ and *β*-PPA. Wang et al. [27] found that the FAZ was not significantly associated with the vessel densities of the retina and choroid in normal subjects. The FAZ has been reported to be enlarged in patients with diabetic retinopathy [28] and retinal vein occlusion [29]. However, the FAZ area did not significantly differ between highly myopic eyes and control group eyes [5] and did not change significantly in response to hyperoxia [30]. This result might also indicate that the regulatory response of the vasculature to hyperoxia differs between the retinal and choroidal circulations, suggesting that the FAZ may not be an ideal location for studying changes in microvessel network density in myopic eyes. Similarly, our study found that the fovea was not correlated with *β*-PPA. This result may be attributable to the thickness of the fovea because the average macular thickness of the foveal and parafoveal regions of myopic patients did not change with the degree of myopia, although the parafovea was thinner and the fovea was thicker due to traction by the vitreous [31]. In addition, measurement of the fovea in OCTA might also have been affected by the FAZ.

There is some strength in this study. First, previous studies demonstrated that vessel density was reduced in high myopia compared with that in healthy subjects. However, these studies did not consider *β*-PPA. To our knowledge, our analysis is the first to study the associations of *β*-PPA with the retinal vasculature in highly myopic eyes. Second, we specially involved young participants with no pathologically myopic change in our study. Third, it is an originally statistical analysis to study the association between *β*-PPA and vessel density in high myopia using a novel quantitative method OCTA. Our findings can increase the clinical significance of *β*-PPA and benefit the early diagnosis and detection of chorioretinal atrophy in high myopia.

There are several potential limitations to the current study. First, this study was limited by its cross-sectional design; in that, the number of participants was not very large. Additional studies with close follow-up of these patients are therefore warranted. Second, to better characterize the relationship between myopia and *β*-PPA, future studies should assess different directional PPA, diffuse PPA, and other types of PPA, such as alpha, gamma, and delta zone and optic disc torsion. Third, we assume that all the eyes are independent from each other in our study; however, there is limitation that some eyes are from same patient. Thus, our findings must be confirmed by further research.

In summary, this study demonstrated a correlation between *β*-PPA and the retinal vasculature in highly myopic eyes using OCTA, thereby presenting a possible reason for the reduced retinal vessel density. However, attention should be given to the AL because when we adjusted for this in the model, the relationship in the macula was not significant. Considering this relationship, retinal fundus photography may be an economic and convenient method for evaluating vessel density in clinical practice, especially for the nasal and inferior sectors. This information is helpful for understanding the pathogenic relationship between myopia and NTG and for the early diagnosis of chorioretinal atrophy in eyes with high myopia. Additionally, *β*-PPA should be adjusted while analysing retinal perfusion using OCTA in future studies.

## Figures and Tables

**Table 1:** Demographic and ocular characteristics of the participants.

	Mean ± SD (*n*=77)	Male (*n*=37)	Female (*n*=40)	*p* value
Age (y)	35.24 ± 8.45	36.29 ± 9.54	34.47 ± 7.55	0.339
SE (D)	10.03 ± 3.57	0.97 ± 0.13	0.98 ± 0.15	0.493
IOP (mmHg)	15.62 ± 3.25	16.60 ± 3.32	14.89 ± 3.03	0.017
*β*-PPA (mm^2^)	1.09 ± 0.62	1.06 ± 0.62	1.10 ± 0.63	0.760
AL (mm)	27.43 ± 1.68	27.67 ± 1.54	27.25 ± 1.77	0.259
SP (mmHg)	119.95 ± 11.90	125.34 ± 12.32	115.94 ± 8.91	<0.001
DP (mmHg)	74.67 ± 8.75	79.43 ± 7.86	71.13 ± 7.70	<0.001
HR	76.11 ± 8.33	74.00 ± 7.23	77.68 ± 8.82	0.047
RNFL (*μ*m)	95.69 ± 0.49	94.58 ± 8.56	96.47 ± 10.11	0.383

SE, spherical equivalent; D, diopters; IOP, intraocular pressure; *β*-PPA, *β*-peripapillary atrophy; AL, axial length; SP, systolic pressure; DP, diastolic pressure; HR, heart rate; RNFL, retinal nerve fiber layer.

**Table 2:** Multivariate regression analysis: association between *β*-PPA and retinal peripapillary capillary (RPC) plexus.

Variable	Model^a^	Unstandardized coefficients		Standardized coefficients	*t*	*p*	Adjusted *R*^2^	95.0% CI for *B*	
		*B*	Std. Error	Beta				Lower bound	Upper bound
*β*-PPA	1	−1.760	0.455	−0.397	−3.871	**<0.001**	**0.147**	−2.665	−0.855
*β*-PPA	2	−1.469	0.475	−0.332	−3.094	**0.003**	**0.172**	−2.414	−0.524
*β*-PPA	3	−1.277	0.438	−0.293	−2.913	**0.005**	**0.305**	−2.150	−0.404
*β*-PPA	4	−1.472	0.459	−0.337	−3.207	**0.002**	**0.313**	−2.386	−0.557

^a^Model 1, crude; model 2, adjustment for age and gender; model 3, further adjustment for intraocular pressure, cup-to-disc ratio, and retinal nerve fiber layer thickness; model 4, further adjustment for axial length.

**Table 3:** Multivariate regression analysis: association between *β*-PPA and deep/superficial retinal plexus.

Variable	Model^a^	Unstandardized coefficients		Standardized coefficients	*T*	*p*	Adjusted *R*^2^	95.0% CI for *B*	
		*B*	Std. Error	Beta				Lower bound	Upper bound
DRP									
*β*-PPA	1	−1.163	0.312	−0.384	−3.722	**<0.001**	0.137	−1.784	−0.541
*β*-PPA	2	−0.874	0.318	−0.289	−2.744	**0.008**	0.202	−1.507	−0.240
*β*-PPA	3	−0.836	0.310	−0.290	−2.697	**0.009**	0.209	−1.454	−0.218
*β*-PPA	4	−0.609	0.318	−0.211	−1.918	0.059	0.251	−1.242	0.024

SRP									
*β*-PPA	1	−1.150	0.403	−0.304	−2.854	**0.005**	0.081	−1.952	−0.348
*β*-PPA	2	−0.924	0.423	−0.244	−2.184	**0.032**	0.099	−1.765	−0.082
*β*-PPA	3	−0.855	0.421	−0.231	−2.029	**0.046**	0.101	−1.694	−0.015
*β*-PPA	4	−0.501	0.427	−0.135	−1.174	0.244	0.181	−1.351	0.350

*β*-PPA, *β*-peripapillary atrophy; DRP, deep retinal plexus; SRP, superficial retinal plexus. ^a^Model 1, crude; model 2, adjustment for age and gender; model 3, further adjustment for intraocular pressure, cup-to-disc ratio, and retinal nerve fiber layer thickness; model 4, further adjustment for axial length.

**Table 4:** Multivariate regression analysis: association between *β*-PPA and macular perfusion parameters in each sector.

Dependent	Model 1	Model 2	Model 3	Model 4
	Coefficient (95% CI)	Coefficient (95% CI)	Coefficient (95% CI)	Coefficient (95% CI)
SRP				
Nasal	**−1.786 (−2.097, −0.600)**	**−1.285 (−2.266, −0.303)**	**−1.274 (−2.270, −0.279)**	−0.868 (−1.877, 0.140)
Temporal	−1.018 (−1.871, −0.166)	−0.725 (−1.622, 0.171)	−0.667 (−1.573, 0.240)	−0.277 (−1.191, 0.637)
Superior	−1.023 (−1.952, 0.093)	−0.777 (−1.766, 0.213)	−0.788 (−1.802, 0.226)	−0.305 (−1.315, 0.705)
Inferior	**−1.669 (−2.799, −0.559)**	**−1.226 (−2.397, −0.058)**	**−1.198 (−2.387, 0.010)**	−0.754 (−1.967, 0.459)
Fovea	−0.019 (−1.150, 1.188)	−0.004 (−1.172, 1.162)	0.216 (−1.120, 1.293)	0.285 (−1.531, 0.962)
FAZ	−0.011 (−0.042, 0.020)	−0.006 (−0.029, 0.018)	−0.078 (−0.031, 0.015)	0.285 (−0.018, 0.028)

DRP				
Nasal	**−1.348 (−2.097, 0.600)**	**−1.043 (−1.817, −0.269)**	**−1.024 (−1.797, 0.252)**	**−1.029 (−1.847, −0.211)**
Temporal	−0.958 (−1.871, −0.166)	**−0.716 (−1.448, 0.015)**	**−0.640 (−1.352, 0.071)**	−0.474 (−1.217, 0.269)
Superior	−0.726 (−1.859, −1.750)	−0.624 (−2.309, 1.061)	−0.598 (−2.330, 1.134)	−0.377 (−2.204, 1.450)
Inferior	**−1.556 (−2.372, 0.741)**	**−1.161 (−1.989, −0.333)**	**−1.125 (−1.969, 0.281)**	−0.746 (−1.592, 0.101)
Fovea	0.281 (−1.12, 1.682)	0.323 (−1.190, 1.836)	0.005 (−1.331, 1.763)	−0.434 (−1.997, 1.130)

Model 1, crude; model 2, adjustment for age and gender; model 3, further adjustment for intraocular pressure, cup-to-disc ratio, and retinal nerve fiber layer thickness; model 4, further adjustment for axial length. *β*-PPA, *β*-peripapillary atrophy; DRP, deep retinal plexus; SRP, superficial retinal plexus.

## Data Availability

All relevant data are included within the article.
